# Maternal Resveratrol Therapy Protects Male Rat Offspring against Programmed Hypertension Induced by TCDD and Dexamethasone Exposures: Is It Relevant to Aryl Hydrocarbon Receptor?

**DOI:** 10.3390/ijms19082459

**Published:** 2018-08-20

**Authors:** Chien-Ning Hsu, Yu-Ju Lin, Pei-Chen Lu, You-Lin Tain

**Affiliations:** 1 Department of Pharmacy, Kaohsiung Chang Gung Memorial Hospital, Kaohsiung 833, Taiwan; chien_ning_hsu@hotmail.com; 2 Department of Obstetrics and Gynecology, Kaohsiung Chang Gung Memorial Hospital and Chang Gung University College of Medicine, Kaohsiung 833, Taiwan; lyu015@cgmh.org.tw; 3 Departments of Pediatrics, Kaohsiung Chang Gung Memorial Hospital and Chang Gung University College of Medicine, Kaohsiung 833, Taiwan; alexiellu@gmail.com; 4 Institute for Translational Research in Biomedicine, Kaohsiung Chang Gung Memorial Hospital and Chang Gung University College of Medicine, Kaohsiung 833, Taiwan

**Keywords:** aryl hydrocarbon receptor, developmental origins of health and disease (DOHaD), hypertension, nitric oxide, oxidative stress, TCDD

## Abstract

Hypertension can originate from early-life adverse environmental in utero exposure to dexamethasone (DEX) or 2,3,7,8-tetrachlorodibenzo-p-dioxin (TCDD). Since DEX and TCDD are related to the aryl hydrocarbon receptor (AHR) signaling pathway, we examined whether resveratrol, an AHR modulator and antioxidant, could prevent programmed hypertension via regulating AHR signaling and oxidative stress. Groups of four-month-old male rat offspring were studied (*n* = 7–8 per group): control, DEX (0.1 mg/kg i.p. from a gestational age of 16 to 22 days), TCDD (200 ng/kg in four once-weekly oral doses), DEX + TCDD, and DEX + TCDD + R (resveratrol 0.05% in drinking water throughout pregnancy and lactation). Maternal TCDD exposure aggravated prenatal DEX-induced hypertension in adult male offspring, which maternal resveratrol therapy prevented. Maternal TCDD exposure aggravated DEX-induced oxidative damage in offspring kidneys, which was prevented by resveratrol therapy. Maternal resveratrol therapy decreased asymmetric and symmetric dimethylarginine (ADMA and SDMA) levels, thereby preventing combined DEX and TCDD exposure-induced programmed hypertension. Increases in renal *Ahrr* and *Cyp1a1* expression induced by DEX + TCDD exposure were restored by resveratrol therapy. The beneficial effects of resveratrol on DEX + TCDD-induced hypertension relate to reduced renal mRNA expression of *Ren*, *Ace*, and *Agtr1a* expression. Thus, the beneficial effects of resveratrol on DEX + TCDD-induced hypertension include reduction of oxidative stress, restoration of nitric oxide (NO) bioavailability, blockade of the renin–angiotensin system (RAS), and antagonizing AHR signaling pathway.

## 1. Introduction

Hypertension is still a global health issue despite recent advances in pharmacotherapies. Hypertension originates from adverse environmental exposures in early life, formally known as the developmental origins of health and disease (DOHaD) [1]. Exposures to environmental chemicals, such as endocrine disrupting chemicals (EDCs), during development can increase the risk of cardiovascular disease later in life [2]. A meta-analysis study of 11 articles reported that EDCs, especially dioxin-related compounds, are associated with the risk of hypertension [3]. In utero exposure to 2,3,7,8-tetrachlorodibenzo-p-dioxin (TCDD), the most toxic member of EDCs, has been reported to increase the vulnerability of offspring to developing hypertension in adulthood [4].

Exposure of the fetus to increased levels of glucocorticoids, resulting from maternal stress or treatment with synthetic glucocorticoids, can cause long-term programmed changes in later life [5]. We previously reported that prenatal exposure to dexamethasone (DEX), a widely used synthetic glucocorticoid, induced programmed hypertension in adult offspring [6,7]. Glucocorticoids have been found to influence the metabolic pathway of TCDD [8]. On the other hand, both glucocorticoids and TCDD can activate the aryl hydrocarbon receptor (AHR) signaling pathway [9,10]. AHR is known to be associated with many different disorders, including hypertension [10]. Progression in the development of hypertension in adulthood can occur with multiple sequential hits. Since each hit induced by prenatal DEX or TCDD exposure may act synergistically to deteriorate programming effects induced by each other, and because DEX and TCDD are closely linked to the AHR pathway, we postulate that maternal TCDD exposure could promote deleterious effects on prenatal DEX-induced renal programming and hypertension in adult offspring via the mediation of the AHR pathway in this two-hit model. 

The kidney is a major organ responsible for blood pressure (BP) control. The developing kidney is vulnerable to adverse early-life environments, which causes long-term morphological and functional changes, namely in renal programming [11]. Some particular mechanisms, such as nitric oxide (NO) deficiency, oxidative stress, and activation of the renin–angiotensin system (RAS), have been studied in terms of renal programming [11,12,13]. Notably, that AHR pathway has been reported to interact with oxidative stress [14] and the RAS [15], two central mechanisms underlying hypertension, to affect BP. However, whether the above-mentioned mechanisms interrelate to induce hypertension of developmental origins in this two-hit model remain unclear. 

Under the DOHaD concept, there may be potential for reprogramming strategies aimed at reversing the programming processes, even before the onset of clinical symptoms, and to thereby shift therapeutic interventions from adulthood to early life [16]. Resveratrol, a natural phytoalexin, has therapeutic potential with a wide range of beneficial effects [17]. Our recent reports demonstrated that resveratrol prevents hypertension of developmental origins induced by combined pre- and post-natal high-fat diets [18]. Given that resveratrol is considered as an AHR modulator [19] and an antioxidant, we thus examined whether maternal resveratrol treatment can protect offspring against combined TCDD and DEX exposure-induced hypertension of developmental origins via the regulation of oxidative stress, NO, RAS, and the AHR signaling pathway. 

## 2. Results

### 2.1. Morphometric Values and Blood Pressures

**Figure 1:**
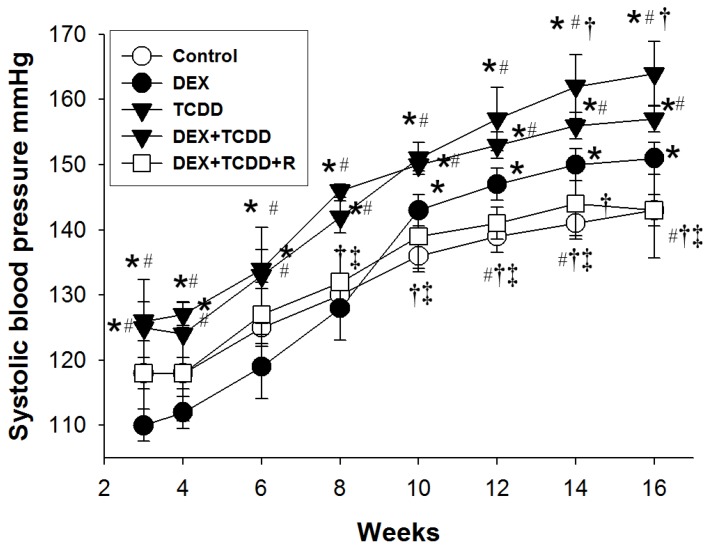
Effects of maternal 2,3,7,8-tetrachlorodibenzo-p-dioxin (TCDD), prenatal dexamethasone (DEX), and resveratrol (R) on systolic blood pressure (SBP) measured in male offspring at 3, 4, 6, 8, 10, 12, 14, and 16 weeks postnatally. Data were analyzed by one-way ANOVA followed by Dunnett’s C post hoc test. * *p* < 0.05 versus control group; ^#^ *p* < 0.05 versus DEX group; ^†^ *p* < 0.05 versus TCDD group; ^‡^ *p* < 0.05 versus DEX + TCDD group (*n* = 7–8/group).

Prenatal DEX or TCDD exposure did not affect the survival of male pups (Table 1). The BW and kidney weight-to-BW ratio were not comparable between the five groups, despite the findings that DEX + TCDD- and DEX + TCDD + R-treated rats had a lower kidney weight compared with vehicle-treated controls. As shown in Figure 1, the systolic blood pressure (SBP) was higher in DEX-treated rats than those in the controls from 10 to 16 weeks of age. After the final dose of TCDD at 2 weeks, the hypertensive effect of TCDD persisted up to 16 weeks in the male offspring. At 16 weeks old, TCDD exposure further increased 13 mmHg of SBP in the DEX + TCDD group compared with that in DEX-treated offspring (Table 1). Resveratrol treatment reduced SBP from 8 to 16 weeks and produced a significant reduction of SBP (~20 mmHg) in DEX + TCDD-exposed male offspring at 16 weeks of age. Similarly, the diastolic BP (DBP) and mean arterial pressure (MAP) of rats exposed to DEX + TCDD was markedly elevated; however, the increases in BP were prevented by maternal resveratrol treatment.

### 2.2. Plasma Levels of l-Cysteine, l-Arginine, ADMA, and SDMA

Since nitric oxide (NO) is a vasodilator and asymmetric dimethylarginine (ADMA)-NO imbalance plays a key role in renal programming and programmed hypertension [20], we then examined the ADMA/NO pathway. As shown in Table 2, combined DEX and TCDD exposure caused a significantly increase in plasma ADMA and symmetric dimethylarginine (SDMA) levels, whereas these increases were attenuated by maternal resveratrol therapy. Additionally, the l-arginine-to-ADMA ratio was the same among the five groups.

### 2.3. Immunohistochemistry Staining of 8-OHdG

**Figure 2:**
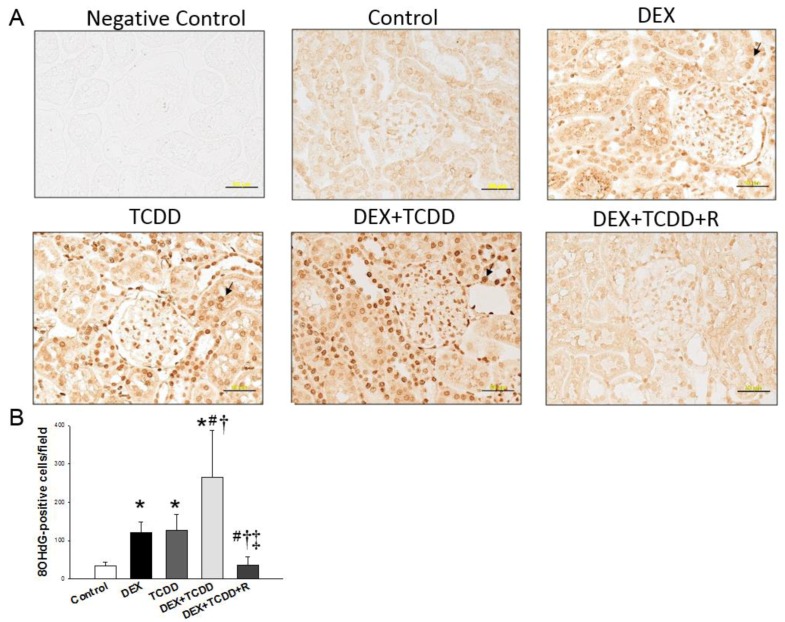
(**A**) Light micrographs illustrating immunostaining for 8-hydroxydeoxyguanosine (8-OHdG) in the kidneys of male offspring at 16 weeks of age. Bar = 50 μm. (**B**) Quantitative analysis of 8-OHdG-positive cells (arrowhead) per microscopic field (×400). Data were analyzed by one-way ANOVA followed by Dunnett’s C post hoc test. * *p* < 0.05 versus control group; ^#^ *p* < 0.05 versus DEX group; ^†^ *p* < 0.05 versus TCDD group; ^‡^ *p* < 0.05 versus DEX + TCDD group (*n* = 5/group).

In order to explore the role of oxidative stress in this two-hit model, we next investigated oxidative stress damage in the kidney by using immunohistochemistry to assay 8-OHdG. The locations of 8-OHdG expression in each renal component are shown in Figure 2A. There were higher expressions of 8-OHdG in the nuclei and cytoplasm of the DEX (120 ± 29 positive cells) and TCDD (126 ± 42 positive cells) groups than in control group (34 ± 9 positive cells). As shown in Figure 2B, there was an even higher 8-OHdG density in the DEX + TCDD group (265 ± 123 positive cells) compared to the controls, which was completely prevented by resveratrol therapy.

### 2.4. Renal mRNA Expression of Genes in AHR Pathway and RAS

**Figure 3:**
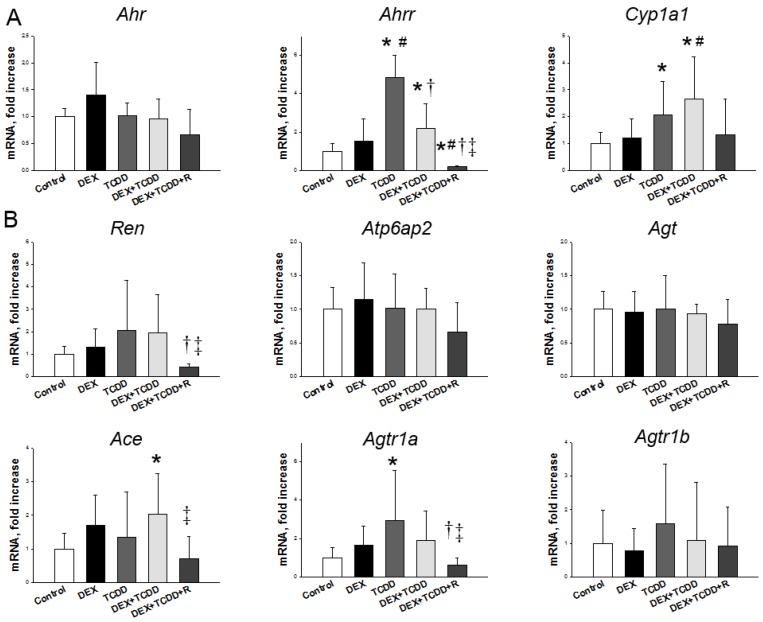
Effects of maternal 2,3,7,8-tetrachlorodibenzo-p-dioxin (TCDD), prenatal dexamethasone (DEX), and resveratrol (R) on mRNA expression of (**A**) aryl hydrocarbon receptor (AHR) signaling pathway, including *Ahr*, *Ahrr*, and *Cyp1a1*; and (**B**) components of renin–angiotensin system (RAS), including *Ren*, *Atp6ap2*, *Agt*, *Ace*, *Agtr1a*, and *Agtr1b* in male offspring kidneys at 16 weeks of age. Data were analyzed by one-way ANOVA followed by Dunnett’s C post hoc test. * *p* < 0.05 versus control group; ^#^ *p* < 0.05 versus DEX group; ^†^ *p* < 0.05 versus TCDD group; ^‡^ *p* < 0.05 versus DEX + TCDD group (*n* = 7–8/group).

Next, we measured the renal mRNA expression of the AHR pathway and RAS components. Renal mRNA expression of *Ahrr* and *Cyp1a1* were higher in TCDD- and DEX + TCDD-treated rats (Figure 3A). Resveratrol treatment significantly reduced *Ahrr* mRNA expression in the DEX + TCDD + R group compared with those in the DEX + TCDD group. We found that TCDD exposure upregulated mRNA expression of *Agtr1a* in the kidney compared with the control group (Figure 3B). Additionally, combined DEX and TCDD exposure increased renal *Ace* mRNA expression in comparison with the controls. The increase in renal *Ace* and *Agtr1a* expression was prevented by resveratrol therapy. In addition, resveratrol therapy downregulated the renal expression of *Ren* in the DEX + TCDD + R group compared with the DEX + TCDD group.

### 2.5. Protein Levels of AHR, AT1R, and AT2R

**Figure 4:**
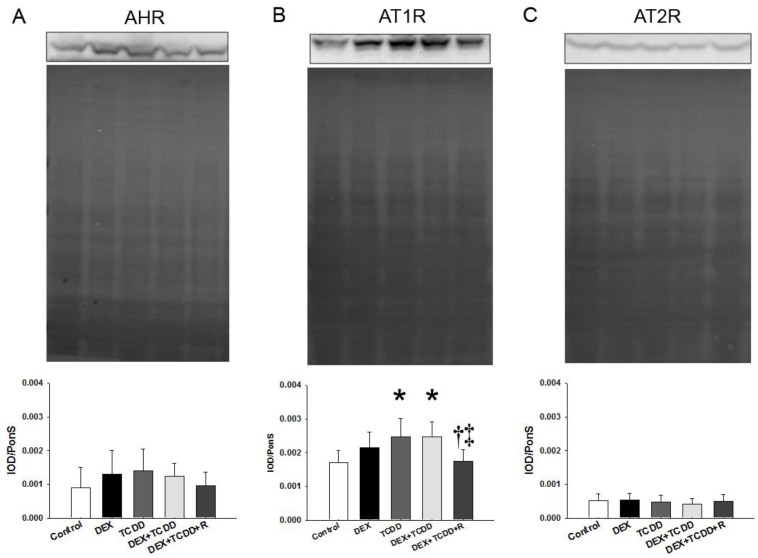
Representative western blots, Ponceau red staining, and relative abundance of (**A**) AHR (96 kDa), (**B**) angiotensin II type 1 (AT1R, 43 kDa), and (**C**) type 2 receptor (AT2R, 50 kDa) in male offspring rats at 16 weeks of age. Data were analyzed by one-way ANOVA followed by Dunnett’s C post hoc test. * *p* < 0.05 versus control group; ^†^ *p* < 0.05 versus TCDD group; ^‡^ *p* < 0.05 versus DEX + TCDD group (*n* = 7–8/group).

Consistent with the change in mRNA level, Figure 4 shows that DEX or TCDD exposure had little measurable effect on the protein levels of AHR and AT2R in offspring kidneys. The renal protein levels of AT1R were increased in the TCDD and DEX + TCDD groups. Resveratrol therapy restored the increase in renal AT1R protein levels in the DEX + TCDD + R group compared to the DEX + TCDD group.

## 3. Discussion

Given the fact that pregnant women have been increasingly exposed to a growing number of environmental stresses and chemicals in the modern world, our study provides novel insights into the beneficial effects of maternal resveratrol treatment of hypertension induced by developmental exposure to TCDD and DEX through mediation of NO, RAS, and the AHR pathway. The major findings are as follows. (1) Maternal TCDD exposure exacerbates prenatal DEX-induced hypertension in adult male offspring; (2) maternal resveratrol therapy prevents combined DEX and TCDD exposure-induced programmed hypertension, which is related to decreased ADMA and SDMA levels; (3) maternal TCDD exposure aggravates DEX-induced oxidative damage in offspring kidneys, which resveratrol therapy prevented; and (4) the beneficial effects of resveratrol on DEX + TCDD-induced hypertension relates to reduced renal mRNA expression of *Ahrr*, *Ren*, *Ace*, and *Agtr1a* expression.

Our data are in agreement with previous studies showing that early exposure to either TCDD or DEX increases the vulnerability of offspring to hypertension in later life [4,5,7]. To our knowledge, this study is the first to show that prenatal DEX and maternal TCDD exposure synergistically induced programmed hypertension in male adult offspring. Our results demonstrated that there was a tendency for combined DEX and TCDD to cause a decrease in BW in adult offspring, despite not reaching statistical significance. Similar to our previous reports [6,7], prenatal DEX exposure had no effect on adult offspring’s BW. Whether combined DEX and TCDD exposure causes a negative effect on BW in adult offspring requires further evaluation. 

Consistent with a number of recent studies which support the importance of oxidative stress relative to programmed hypertension [11,12,13], our data demonstrate that maternal exposure to DEX and TCDD induces oxidative stress and hypertension concurrently. TCDD has been reported to increase BP via increased renal oxidative stress in adult rats [19]. Our observations provide further evidence that a combination of TCDD and DEX exposure has a synergistic effect on oxidative stress damage. It is possible that a greater degree of oxidative stress contributes to an exacerbation of programmed hypertension.

Oxidative stress is an oxidative shift characterized by dysregulation of NO and reactive oxygen species (ROS). ADMA is considered a major player in causing a NO–ROS imbalance [21]. The data presented here show that a combination of TCDD and DEX exposure induces an increase in ADMA and SDMA levels and a decrease in the l-arginine-to-ADMA ratio. Like ADMA, SDMA can inhibit NO production [22]. Since the l-arginine-to-ADMA ratio has been used to represent NO bioavailability [23], our findings imply that combined TCDD and DEX exposure increases ADMA and SDMA, consequently decreasing NO bioavailability, and may be a major mechanism underlying hypertension of developmental origins. 

In addition to their effects on oxidative stress, combined TCDD and DEX exposure were also found to activate the RAS. The classical RAS, defined as the ACE-angiotensin (Ang) II-AT1R axis, promotes vasoconstriction and sodium retention, leading to hypertension. In agreement with a previous study showing that perinatal TCDD exposure increases the susceptibility of offspring to Ang II-induced hypertension [4], we observed that hypertension induced by TCDD exposure related to increased renal *Ace* and *Agtr1a* mRNA expression. Consistent with growing evidence that early blockade of RAS in young offspring protects against the development of hypertension [24], the data presented here support the view that maternal resveratrol therapy reduces renal *Ren*, *Ace*, and *Agtr1a* expression and thereby blocks the RAS to provide protection against the development of hypertension.

Resveratrol, a natural phytoalexin, has found uses in a broad spectrum of clinical applications, including in the treatment of hypertension [25]. It has been used to prevent detrimental effects induced by developmental exposure to TCDD [26]. However, little is known about the protective effects of maternal resveratrol treatment of hypertension induced by in utero TCDD exposure. Our recent report showed that resveratrol prevents hypertension of developmental origins induced by combined pre- and post-natal high-fat consumption via medication of oxidative stress, NO, and the RAS [18]. This notion is corroborated by our current study, suggesting that the protective effects of maternal resveratrol treatment on DEX + TCDD-induced hypertension are related to a reduction of oxidative stress, increased NO bioavailability, and a blockade of the RAS.

An additional protective mechanism of resveratrol on programmed hypertension in this two-hit model may be related to mediation of the AHR signaling pathway. AHR signaling can be activated by exogenous ligand TCDD to target AHR gene expression, such as *Ahrr* and *Cyp1a1* [27]. Significantly increased mRNA expression of *Ahrr* and *Cyp1a1* in the kidneys of TCDD-exposed rats indicates AHR activation, which appears to be crucial in BP control [10]. Conversely, increases in renal *Ahrr* and *Cyp1a1* expression induced by DEX + TCDD exposure were restored by resveratrol therapy. Although glucocorticoid has been reported to induce AHR and its target genes in adult rats [9], this notion is not supported by the present observations, which showed that prenatal DEX exposure had no effect on the AHR signaling pathway in offspring. These and previous observations suggest that resveratrol may act as an AHR antagonist and inhibit TCDD-induced AHR target gene expression [19,26]. As such, whether other AHR antagonists can provide a therapeutic approach to prevent hypertension of developmental origins requires further study and clarification.

Our study has a few limitations worth noting. Although we focus on the kidney in the present study, the protective effect of resveratrol may be attributed to other organs that control BP, such as the vasculature, the heart, and the brain. Additionally, whether NO deficiency, oxidative stress, activation of the RAS, and AHR signaling in other organs and tissues contribute to programmed hypertension requires further clarification. Secondly, we restricted resveratrol therapy to the DEX + TCDD group because resveratrol has no effect on BP in normotensive controls [28] and the effect of resveratrol therapy on TCDD-exposed offspring has been studied [26]. Moreover, previous studies in humans have already shown that the bioavailability of resveratrol after oral intake is rather low [29]. The development of resveratrol formulations with better biotransformation and pharmacologic properties is still a challenging task for clinical translation in the future. Lastly, we did not explore different doses or exposure times for TCDD; given that most exposures are polychemical and dose-dependent, programming effects may vary during different types of developmental exposures.

To summarize, TCDD exposure exacerbates prenatal DEX-induced hypertension of developmental origins in adult male offspring. Several important mechanisms by which maternal resveratrol therapy protects offspring against combined TCDD and DEX exposure-induced programmed hypertension reduce oxidative stress, increase NO bioavailability, block the RAS, and antagonize AHR signaling. It is clear that a better understanding of the types of chemicals, exposure doses, critical windows, and therapeutic durations for reprogramming interventions are required to help pregnant women and their children against increasing environmental stresses and chemicals.

## 4. Materials and Methods 

### 4.1. Animal Models

This study was approved by the Institutional Animal Care and Use Committee of the Kaohsiung Chang Gung Memorial Hospital (Permit number: 2017031602, 16 May 2017). Virgin Sprague-Dawley (SD) rats (12–16 weeks old) were obtained from BioLASCO Taiwan Co., Ltd. (Taipei, Taiwan). Animals were maintained in an AAALAC-accredited facility, housed in a 12 h light/12 h dark cycle with ad libitum access to tap water and standard rat chow. Animal care and use was in strict accordance with the recommendations of the Guide for the Care and Use of Laboratory Animals of the National Institutes of Health. Male SD rats were housed with individual females until mating was confirmed by the examination of a vaginal plug. Since hypertension occurs at a higher rate and at an earlier age in males than females [30], only male offspring were selected from each litter and used in subsequent experiments. After their birth, litters were culled to a total of eight pups to standardize the received quantity of milk and maternal pup care. Male offspring were assigned to five groups (*n* = 7–8 for each group): control, DEX, TCDD, DEX + TCDD, and DEX + TCDD + R.

To construct a prenatal DEX exposure model, dexamethasone (0.1 mg/kg body weight [BW]; Taiwan Biotech Co., Ltd., Taoyuan, Taiwan) or vehicle was intraperitoneally administered to pregnant SD rats from a gestational age of 16 to 22 days [6,7]. Additionally, pregnant dams received TCDD (200 ng/kg BW orally; Sigma-Aldrich, St. Louis, MO, USA) or corn oil vehicle (4 mL/kg BW) on gestational days 14 and 21 and postnatal days 7 and 14 to provide in utero and lactation exposure of pups to the dioxin, respectively. The weekly dose of TCDD used here was based on previous studies because TCDD has a long half-life of ~3 weeks in rats [31,32]. In addition to DEX and TCDD exposure, mother rats in the DEX + TCDD + R group received resveratrol 0.05% in drinking water during the pregnancy and lactation period [33].

BP was measured in conscious rats in weeks 3, 4, 6, 8, 10, 12, 14, and 16 by using an indirect tail-cuff method (BP-2000, Visitech Systems, Inc., Apex, NC, USA) as previously described [7]. To ensure accuracy and reproducibility, the rats were allowed to adapt to restraint and tail-cuff inflation for 1 week prior to the experiment, and measurements were taken in the afternoon each day. Rats were placed on a specimen platform, and their tails were passed through tail cuffs and secured with the tape. Following a 10-min warm-up period, 10 preliminary cycles of tail-cuff inflation were performed to allow the rats to adjust to the inflating cuff. For each rat, five cycles were recorded at each time point. Among them, three stable consecutive measures were taken and averaged. All offspring were killed at 16 weeks of age. Rats were anesthetized using an intraperitoneal injection of ketamine (50 mg/kg) and xylazine (10 mg/kg), then euthanized by an intraperitoneal overdose of pentobarbital. Heparinized blood samples were collected at the end of the study. The kidneys were removed and divided into cortex and medulla and snap frozen at −80 °C for further analysis.

### 4.2. High-Performance Liquid Chromatography (HPLC)

The levels of several components of the NO pathway, including l-citrulline, l-arginine, ADMA, and SDMA (an isomer of ADMA), were measured using HPLC (HP series 1100; Agilent Technologies Inc., Santa Clara, CA, USA) with the *O*-phtalaldehyde-3-mercaptoprionic acid derivatization reagent [7]. Standards contained concentrations of 1–100 mM l-citrulline, 1–100 mM l-arginine, 0.5–5 mM ADMA, and 0.5–5 mM SDMA. 

### 4.3. Quantitative Real-Time Polymerase Chain Reaction

RNA was extracted using TRIzol reagent, treated with DNase I (Ambion, Austin, TX, USA) to remove DNA contamination, and reverse transcribed using random primers (Invitrogen, Carlsbad, CA, USA). Control RT reactions were performed by omitting RT enzyme. RNA concentration and quality were checked by measuring optical density at 260 and 280 nm. Complementary DNA was synthesized using M-MLV Reverse Transcriptase (Invitrogen). Two-step quantitative real-time polymerase chain reaction (PCR) was performed using QuantiTect SYBR Green PCR Kit (Qiagen, Valencia, CA, USA) and iCycler iQ Multi-Color Real-Time PCR Detection System (Bio-Rad, Hercules, CA, USA). Three genes involved in the AHR signaling pathway were analyzed, including *Ahr*, *Ahrr* (encoded for aryl hydrocarbon receptor repressor), and *Cyp1a1* (Cytochrome P450 CYP1A1). Components of the RAS were analyzed including *Ren* (encoded for renin), *Atp6ap2* (encoded for prorenin receptor), *Agt* (angiotensinogen), *Ace* (angiotensin-converting enzyme), *Agtr1a*, and *Agtr1b* (encoded for angiotensin II type 1 and 2 receptor) [31]. *Rn18s* (18S rRNA) was used as a reference gene. The sequences of the primers used in this study are provided in Table 3. Primer efficiency between 1.8 and 2.2 was acceptable. Comparative threshold (*C*t) values were used for all manipulations and were first normalized to average the *Rn18s* value by calculating the Δ*C*t for each sample. Values were then calculated relative to controls to generate a ΔΔ*C*t value. The fold-increase of the experimental sample relative to the control was calculated using the formula 2^−ΔΔ*C*t^.

### 4.4. Western Blotting

Western blot analysis was performed as previously described [7]. We used the following antibodies: for AHR, a rabbit anti-rat AHR antibody (1:1000, overnight incubation; NB100-2289, Novus Biologicals, Littleton, CO, USA); for angiotensin II type 1 receptor (AT1R), a rabbit anti-rat AT1R antibody (1:500, overnight incubation; AB15552, Millipore, Billerica, MA, USA); for angiotensin II type 2 receptor (AT2R), a rabbit anti-rat AT2R antibody (1:250, overnight incubation; sc-9040, Santa Cruz Biotechnology, Santa Cruz, CA, USA). Bands of interest were visualized using enhanced chemiluminescence reagents (PerkinElmer, Waltham, MA, USA) and quantified by densitometry (Quantity One Analysis software; Bio-Rad), as integrated optical density (IOD) after subtraction of background. The IOD was factored for Ponceau red staining to correct any variations in total protein loading. The protein abundance was represented as IOD/PonS.

### 4.5. Immunohistochemistry Staining

Paraffin-embedded tissues sectioned at-μm thickness were deparaffinized in xylene and rehydrated in a graded ethanol series to phosphate-buffered saline. 8-Hydroxydeoxyguanosine (8-OHdG) is a DNA oxidation product that was measured to assess DNA damage. Following blocking with immunoblock (BIOTnA Biotech., Kaohsiung, Taiwan), the sections were incubated for 2 h at room temperature with an anti-8-OHdG antibody (1:100, JaICA, Shizuoka, Japan). A negative control of identical staining omitting incubation with a primary antibody was used. Immunoreactivity was revealed using the polymer-horseradish peroxidase (HRP) labeling kit (BIOTnA Biotech) and, 3′-diaminobenzidine (DAB) as the chromogen. 8-OHdG-positive cells per microscopic field (×400) in the renal sections were quantitatively analyzed as previously described [34].

### 4.6. Statistical Analysis

The values given in the figures and tables represent the mean ± standard deviation. Statistical analysis was done using one-way ANOVA with Dunnett’s C post hoc test. In all cases, a *p*-value of 0.0 was considered statistically significant. All analyses were performed using the Statistical Package for the Social Sciences software (SPSS, Chicago, IL, USA).

## Figures and Tables

**Table 1:** Weight and Blood Pressure.

	Control	DEX	TCDD	DEX + TCDD	DEX + TCDD + R
	*n* = 7	*n* = 8	*n* = 8	*n* = 8	*n* = 8
Mortality	0%	0%	0%	0%	0%
Body weight (BW) (g)	532 ± 62	526 ± 60	523 ± 29	470 ± 30	512 ± 87
Left kidney weight (g)	2.08 ± 0.18	1.91 ± 0.29	1.8 ± 0.16	1.75 ± 0.15 ^a^	1.73 ± 0.18 ^a^
Left kidney weight/100 g BW	0.39 ± 0.04	0.36 ± 0.03	0.34 ± 0.02	0.38 ± 0.05	0.34 ± 0.04
Systolic blood pressure (mmHg)	143 ± 3	151 ± 3 ^a^	158 ± 4 ^a^	164 ± 6 ^a,b^	143 ± 9 ^b,c,d^
Diastolic blood pressure (mmHg)	66 ± 6	63 ± 5	70 ± 3	76 ± 5 ^b^	69 ± 11
Mean arterial pressure (mmHg)	92 ± 5	92 ± 3	100 ± 3 ^a,b^	106 ± 4 ^a,b,c^	94 ± 9

Data were analyzed by one-way ANOVA followed by Dunnett’s C post hoc test. ^a^ *p* < 0.05 versus control group; ^b^ *p* < 0.05 versus DEX group; ^c^ *p* < 0.05 versus TCDD group; ^d^ *p* < 0.05 versus DEX + TCDD group.

**Table 2:** Measures of Plasma l-Citrulline, l-Arginine, and Dimethylarginine Levels.

	Control	DEX	TCDD	DEX + TCDD	DEX + TCDD + R
	*n* = 7	*n* = 8	*n* = 8	*n* = 8	*n* = 8
l-Citrulline (μM)	32.9 ± 4.8	32.5 ± 5.3	28.9 ± 6.0	30.8 ± 2.3	16 ± 9.2
l-Arginine (μM)	129.3 ± 30.6	150.5 ± 47.9	120.7 ± 27.7	130.1 ± 24.3	146.8 ± 35.5
ADMA (μM)	0.24 ± 0.09	0.73 ± 0.09 ^a^	0.6 ± 0.33	1.04 ± 0.59 ^a^	0.44 ± 0.89 ^b^
SDMA (μM)	0.08 ± 0.04	0.23 ± 0.52 ^a^	0.14 ± 0.55	0.58 ± 0.54 ^a^	0.26 ± 0.55
l-arginine-to-ADMA ratio (μM/μM)	587 ± 221	203 ± 53	246 ± 108	203 ± 181	336 ± 84

Data were analyzed by one-way ANOVA followed by Dunnett’s C post hoc test. ^a^ *p* < 0.05 versus control group; ^b^ *p* < 0.05 versus DEX group.

**Table 3:** PCR Primer Sequences.

Gene	Forward (5′–3′)	Reverse (5′–3′)
*Ahr*	gtcctcagcaggaacgaaag	ccagggaagtccaactgtgt
*Ahrr*	cagcaacatggcttctttca	tgaagcactgcattccagac
*Cyp1a1*	gcactctggacaaacacctg	atatccaccttctcgcctgg
*Ren*	aacattaccagggcaactttcact	acccccttcatggtgatctg
*Atp6ap2*	gaggcagtgaccctcaacat	ccctcctcacacaacaaggt
*Agt*	gcccaggtcgcgatgat	tgtacaagatgctgagtgaggcaa
*Ace*	caccggcaaggtctgctt	cttggcatagtttcgtgaggaa
*Agtr1a*	gctgggcaacgagtttgtct	cagtccttcagctggatcttca
*Agtr1b*	caatctggctgtggctgactt	tgcacatcacaggtccaaaga
*Rn18s*	gccgcggtaattccagctcca	cccgcccgctcccaagatc

*Ahr* = Aryl hydrocarbon receptor, *Ahhr* = Aryl hydrocarbon receptor repressor, *Cyp1a1* = Cytochrome P450 CYP 1A1, *Ren* = Renin, *Atp6ap2* = Prorenin receptor (PRR), *Agt* = Angiotensinogen (AGT), *Ace* = Angiotensin converting enzyme (ACE), *Agtr1a* = Angiotensin II type 1 receptor (AT1R), *Agtr1b* = Angiotensin II type 2 receptor (AT2R), *Rn18s* = 18S ribosomal RNA (r18S).

## Abbreviations

ACE	Angiotensin-converting enzyme
ADMA	Asymmetric dimethylarginine
AHR	Aryl hydrocarbon receptor
AT1R	Angiotensin II type 1 receptor
AT2R	Angiotensin II type 2 receptor
DEX	Dexamethasone
DOHaD	Developmental origins of health and disease
EDC	Endocrine disrupting chemical
NO	Nitric oxide
RAS	Renin–angiotensin system
SDMA	Symmetric dimethylarginine
TCDD	2,3,7,8-tetrachlorodibenzo-p-dioxin
8-OHdG	8-Hydroxydeoxyguanosine
